# Impact of Plant Extract Phytochemicals on the Synthesis of Silver Nanoparticles

**DOI:** 10.3390/ma17102252

**Published:** 2024-05-10

**Authors:** Oksana Velgosova, Silvia Dolinská, Helena Podolská, Lívia Mačák, Elena Čižmárová

**Affiliations:** 1 Institute of Materials and Quality Engineering, Faculty of Materials Metallurgy and Recycling, Technical University of Košice, Letná 9/A, 042 00 Košice, Slovakia; helena.podolska@student.tuke.sk (H.P.); livia.macak@tuke.sk (L.M.); 2 Institute of Geotechnics, Slovak Academy of Sciences, Watsonova 45, 040 01 Košice, Slovakia; sdolinska@saske.sk; 3 Department of Materials Engineering, Faculty of Mechanical Engineering, Czech Technical University in Prague, Karlovo nám. 13, 121 32 Prague, Czech Republic; elena.cizmarova@fs.cvut.cz

**Keywords:** AgNPs, green synthesis, functional groups, FTIR, TEM

## Abstract

This work aims to analyze the influence of selected plants on the synthesis of silver nanoparticles (AgNPs). Six plants were chosen for the experiment, from which extracts were prepared: maclura fruit, spruce and ginkgo needles, green algae (*Ch. kessleri*), and mushrooms, namely *Collybia nuda*, and *Macrolepiota procera*. The composition of the extracts and colloids after preparation of the nanoparticles was analyzed using FTIR analysis. The composition of the extracts affected not only the rate of the synthesis but also the shape of the nanoparticles. TEM analysis confirmed the synthesis of mainly spherical nanoparticles (size range: 10–25 nm). However, triangular prisms and polyhedral nanoparticles synthesized by the extracts containing mainly flavonoids, terpenes, and phenols (the main compounds of resins) were also confirmed. EDS analysis was used to analyze the composition of the nanoparticles. It was proven that by choosing the right plant extract and using the appropriate technology with extract treatment, it is possible to prepare nanoparticles of different shapes.

## 1. Introduction

One of the greatest benefits of nanotechnology is its ability to create materials with unique properties that cannot be achieved with classical technologies. Nanoscale materials can be more durable and lighter or have improved electrical, optical, or mechanical properties [1], and thanks to these unique properties, they have massive potential in almost every field of industry.

The two main approaches to producing nanoparticles are bottom-up and top-down. These approaches differ in their manufacturing process and the associated quality, speed, and cost. The bottom-up method enables efficient synthesis of nanoparticles through controlled chemical reactions. It is a simple and inexpensive method that controls the size, shape, and other nanoparticle properties, which is crucial for many applications. Currently, much attention is directed toward so-called green synthesis, which represents a significant advance over classical chemical synthesis. In this method, biological reducing agents replace the toxic agents, significantly moving the green method to the forefront due to economic and environmental advantages [2,3,4]. The economic advantage of green methods mainly leads to favoring them over physical ones, which require expensive, energy-intensive equipment. Compared with chemical methods, they are also more advantageous because they do not require the purchase of expensive chemicals. It is possible to argue that, for example, when using bacteria, the process becomes more expensive due to the necessity of their cultivation, and at intracellular synthesis, it is necessary to extract nanoparticles from the cells, which makes the process more expensive. However, these disadvantages are solved by synthesis using plant extracts. Extracts obtained from various biomaterials, such as plants, fruits, fungi, alga, and various microorganisms, are often used as reducing and stabilizing agents. Extracts from different species can have different amounts and types of phytochemicals, as well as different concentrations of biologically active compounds like proteins, polysaccharides, lipids, and nucleic acids, which subsequently affect the synthesis [5].

In the work of Chugh et al., a review of synthesized nanoparticles using algae was presented. Three types of alga were used in this research: cyanobacteria, microalgae, and macroalgae, each of which included four specific species. An interesting finding from this study was that despite all these species belonging to the same family, the results of the nanoparticle synthesis varied greatly. The differences were related not only to the resulting sizes of the nanoparticles but, in some cases, also to their shapes [6]. Although the exact mechanism and function of the functional groups in the bio-compounds involved in the synthesis are not fully known, most authors try to describe, based on the results of Fourier transform infrared spectroscopy (FTIR), which functional groups are responsible for reduction and stabilization of silver nanoparticles.

The infrared spectroscopy method allows us to identify the compositions of biomolecules by analyzing their functional groups. FTIR measures the vibrations and rotations of molecules induced by infrared radiation, which makes it possible to reveal the structural differences and interactions between substances. This method is useful for identifying an extract’s composition and comparing it with FTIR analysis of the resulting colloidal solution [7].

Several authors devoted themselves to monitoring the influence of individual compounds on the synthesis of AgNPs. For example, Mohanta et al. used an extract from the mushroom *Ganoderma applanatum* to synthesize silver nanoparticles. The resulting nanoparticles had an average size of ~133 nm. Using FTIR analysis, the authors studied the functional groups and confirmed that mainly hydroxyl and carbonyl groups are responsible for the synthesis of AgNPs [8]. Singh et al. described the influence of phenolic compounds, terpenoids, and proteins on the bioreduction of metal ions and the stabilization of metal nanoparticles obtained from plant extracts. Phenolic compounds, such as phenolic acid, support antioxidant activity and metal chelation, thereby enabling the reduction and formation of stabilized nanoparticles. Terpenoids such as eugenol, obtained from essential oils of plants, serve as a bioreducing agent, with their homolytic breakdown upon exposure to sunlight allowing the formation of metal nanoparticles. Proteins and amino acids, especially those with exposed disulfide bonds and thiols, function as reducing agents and stabilizers during the biosynthesis of nanoparticles [9].

In the field of nanoparticle synthesis, there is a plentiful selection of biomaterials that can be explored. Various combinations of extracts and methods of extract preparation offer wide possibilities for achieving the desired properties of nanoparticles. The composition of the extract depends mainly on the plant from which it is prepared. Not only the type of plant and genus (higher plants, fungi, algae, etc.) but also its parts (fruits, leaves, roots, seeds, etc.) have different compositions. Plants can contain flavonoids, proteins, carbohydrates, antioxidants, vitamins, and lipids, but some also contain different essential oils and resins. The presence of these substances or their combination can affect the shape and size of nanoparticles as well as the yield of the process.

In our study, we focused on studying the influence of six different types of biomaterials on AgNPs synthesis. We chose biomaterials that we assumed would have significantly different compositions: Maclura fruits, edible forest mushrooms, needles (spruce and ginkgo), and unicellular algae. Extracts prepared from the selected plants were used as a reducing agent. We monitored the differences in the compositions of the extracts (using FTIR analysis) and the influence of the extract composition on the synthesis of nanoparticles, namely through the synthesis rate, shape, and size of the resulting nanoparticles. The obtained results can shed light on the optimization of the process of nanoparticle synthesis using plant extracts.

## 2. Materials and Methods

### 2.1. Materials

As a precursor of silver ions, silver nitrate (AgNO_3_, >98%) purchased from Mikrochem Ltd. in Pezinok, Slovakia was used. Different types of biological materials—*Maclura pomifera* fruit (maclura), *Ginkgo biloba* leaves (ginkgo), green alga *Chlorella kessleri* (*Ch. kessleri*), *Picea abies* needles (spruce needles), *Collybia nuda* (wood blewit), and *Macrolepiota procera* (parasol mushroom)—were used as sources of reducing and stabilizing agents. All biological materials were collected in a local botanical garden in Košice, Slovakia or an area near Košice. Deionized water was used for preparing all solutions.

### 2.2. Preparation of Extracts

The biological materials, except for the *Ginkgo biloba* leaves, were used dry. We used the same weight of biomaterials (2.5 g), but after drying, their weights differed (Table 1). Before preparing the extract, all plants and fruits were weighed, crushed in a mortar, and poured into a 50 mL beaker with deionized water. The quantities of the used compounds are listed in Table 1. The mixtures were heated in a water bath with constant stirring for 15 min. Subsequently, all extracts were cooled to ambient temperature and filtered using filter paper. 

Extracts of the macula, ginkgo, spruce, *Ch. kessleri*, and both mushrooms after filtration were centrifuged at 9000 rpm for 10 min to remove the solid phase from the extracts thoroughly. Subsequently, the pH levels of all extracts were measured. The extracts were used immediately after preparation.

### 2.3. Synthesis of AgNP Colloids

The same procedure was used to prepare colloidal solutions of silver nanoparticles (6 colloids), and the procedure was as follows:

- First, 50 mL of AgNO_3_ stock solution (concentration of 50 mg/L, prepared by the dissolution of AgNO_3_ powder in deionized water) was heated to 80 °C in a water bath with constant stirring.
- Subsequently, 5 mL of extract was added dropwise to the stock solution, and the prepared mixture was kept at a temperature of 80 °C for 15 min with constant stirring.
- The prepared colloids were cooled and analyzed by UV-vis spectrophotometry, and the pH levels of the colloids were also measured. All colloids were stored at room temperature.

UV-vis analysis requires a control sample. For each AgNP colloidal solution, the controls were prepared in the same ratio as the nanoparticle colloids: 5 mL of water and 0.5 mL of extract. Control samples were stored in the refrigerator to avoid changes in their content. UV-vis measurements were made after colloid preparation (D0) and on the first, fourth, seventh, and fourteenth days (D1, D4, D7, and D14, respectively). All experiments were repeated four times.

### 2.4. Methods and Analysis

The prepared solutions were analyzed with a UV-vis spectrophotometer (UNICAM UV-vis Spectrophotometer UV4). The size and morphology of the nanoparticles were studied using TEM (JEOL model JEM-2000FX microscope operated at an accelerating voltage of 200 kV, JEOL, Tokyo, Japan). The image analysis ImageJ 1.54g software was used for the analysis of the AgNP size distribution. XRD analysis was used to demonstrate the silver in the nanoparticles.

The infrared spectra were recorded with a Bruker Tensor 27 FTIR spectrometer (Bruker, Billerica, MA, USA) equipped with a DTGS KBr detector. For each sample, 64 scans were measured in the 4000–400 cm^−1^ spectral range in abs mode with a resolution of 4 cm^−1^. The KBr pressed-disc technique was used to prepare a solid sample for routine spectra scanning. Samples of approximately 0.1 mg were dispersed in 150 mg of KBr to record optimal spectra in the regions of 4000–400 cm^−1^. The diameter of the pellets pressed from the samples was 13 mm.

## 3. Results

### 3.1. Analysis of Extract

**Figure 1:**
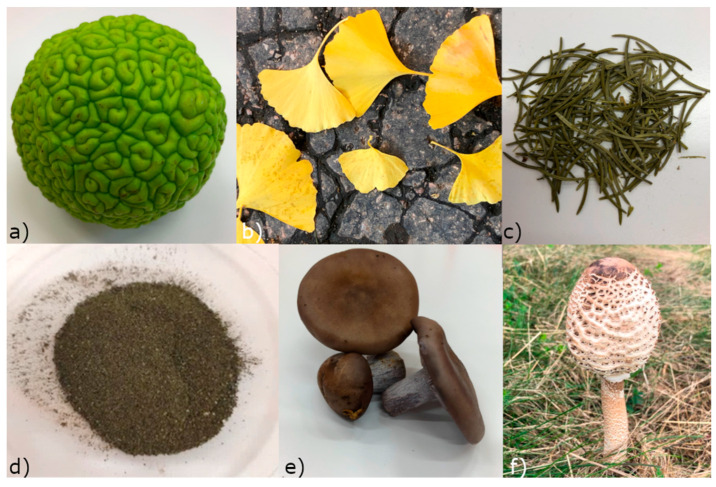
Biological material used for preparation of extracts: maclura (**a**), leaves of ginkgo (**b**), spruce needles (**c**), *Ch. kessleri* (**d**), *Collybia nuda* (**e**), and *Macrolepiota procera* (**f**).

Figure 1 shows the biological material that was used for the synthesis of silver nanoparticles. Biological material naturally contains compounds that can reduce Ag^+^ ions to zero-valent silver Ag^0^ and at the same time also contain effective stabilizing agents.

The biological material used was chosen in such a way as to ensure the diversity of the composition. For example, in the case of ginkgo, it is mentioned that the main medicinal constituents of ginkgo are found in the leaf. Ezzat H. et al. determined that bioactive compounds include flavonoid glycosides, such as myricetin, kaempferol, isorhamnetin, and quercetin, and the major terpene molecules are ginkgolides and bilobalide [10,11,12]. Approximately 40 minor flavonoids were identified in ginkgo [13]. Other leaf compounds include the steroid sitosterol, polyprenols, carbohydrates, alcohol, ketones, and different acids (vanillic and ascorbic). It was proven that there is seasonal variation in the active compound content of the leaves, with the highest amounts present in autumn [13]. Therefore, the leaves for the experiment were collected in October.

*Maclura pomifera* fruit contains some phytochemicals which may include flavonoids (quercetin, kaempferol, and rutin). Also, polyphenols are another group of antioxidants found in plants (resveratrol, ellagic acid, and catechins), terpenoids, carotenoids (β-carotene, lutein, and zeaxanthin), vitamins (C and A), minerals (potassium), and fiber. It is important to note that maclura fruit has thus far been researched little, and further research is needed to understand its specific composition and potential health benefits fully.

Spruce needles contain a variety of phytochemicals which contribute to the medicinal and nutritional properties of spruce needles. They also contain vitamin C, ascorbic acid, which is a powerful antioxidant, flavonoids (quercetin, kaempferol, and rutin), terpenes (α-pinene, β-pinene, limonene, and bornyl acetate), tannins, polyphenolic compounds, and different essential oils.

*Ch. kessleri* is a green microalga rich in proteins which contains essential amino acids. *Ch. kessleri* also contains polysaccharides, carbohydrates, polyphenols, carotenoids, phycobiliproteins, vitamins, and sterols, as well as polyunsaturated fatty acids such as linoleic acid and alpha-linolenic acid [14,15,16].

*Collybia nuda* (wood blewit) contains minerals, especially potassium, proteins, and folic acid, as well as flavonoids, fatty acids, polysaccharides, and antibacterial and antioxidant substances.

*Macrolepiota procera* (parasol mushroom) is an edible mushroom. Therefore, it contains various nutrients such as proteins, carbohydrates, fiber, and minerals. They also contain vitamins such as vitamin D. In addition, aromatic compounds, aldehydes (octanal-acetic aldehyde), phenols (guaiacol and lentic acid), esters (ethyl acetate), and thioesters were confirmed.

**Figure 2:**
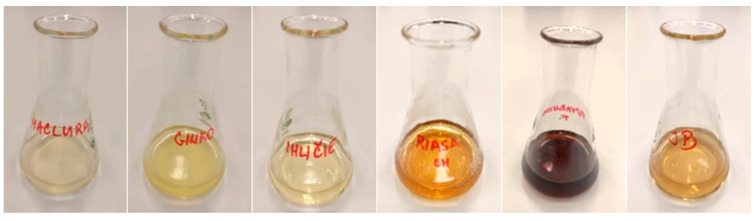
Extracts prepared from biological materials: maclura, ginkgo, spruce needles, *Ch. kessleri*, *Collybia nuda*, and *Macrolepiota procera* (from left to right).

The process of nanoparticle synthesis depends on several factors, with the most important one being the extract (its composition), but pH [17,18] also has a fundamental influence [19,20,21]. Various pH values can alter the ionization of functional groups in biomaterials or the chemical components used in the synthesis, leading to changes in the reduction process of silver ions and the formation of AgNPs. Additionally, pH variation can impact the stability and agglomeration of AgNPs, as it affects the electrostatic interactions between particles and their surroundings [18]. Figure 2 shows the extracts prepared using biological materials.

**Figure 3:**
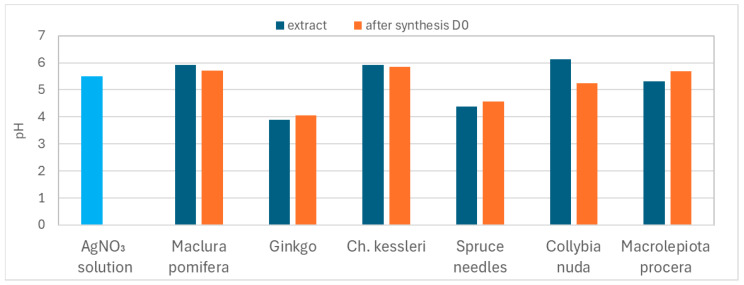
The pH values of AgNO_3_ solution (blue bar) and extracts and the pH values of prepared colloids on D0.

Figure 3 shows a bar graph of the pH values of the AgNO_3_ solution, all prepared extracts, and the colloidal solutions after synthesis (on D0). The pH level differed depending on the type of plant, and several authors have confirmed that pH affects the reducing activity of plant extracts [17,18]. The extract prepared from ginkgo and needles had the lowest pH, with the others ranging from 5 to 6.

The critical pH value below which nanoparticles are not formed is approximately 3.5. Several authors agree that nanoparticles form faster in an alkaline environment than in an acidic environment [19], and those synthesized in an acidic environment are lower than those in an alkaline environment [20,21]. This could be accredited to the ionization of the functional groups at higher pH values, and the slow rate of reduction observed in the acidic medium could be attributed to the electrostatic repulsion of anions present in the reaction mixture [21]. It is obvious from Figure 3 that after the formation of AgNPs, there was no significant change in the pH values of the colloidal solutions.

**Figure 4:**
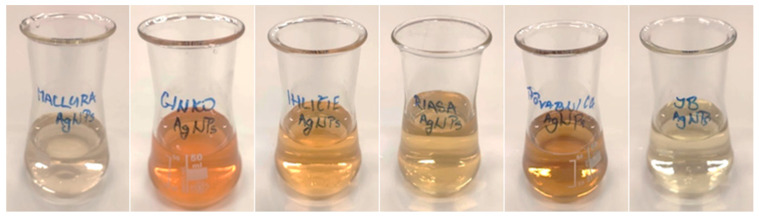
AgNP colloids prepared by biological method after synthesis on D0: maclura, ginkgo, spruce needles, *Ch. kessleri*, *Collybia nuda*, and *Macrolepiota procera* (from left to right).

After adding the individual extracts to the silver precursor solution (Figure 2), the reduction reactions started, which caused a change in color for all solutions (Figure 4). A color change is the first sign of successful synthesis of silver nanoparticles. The more intense the coloring, the more nanoparticles there are in the solution. It is clear that the rate of the reduction process was not the same in all extracts, which was also confirmed by UV-vis spectrophotometry.

### 3.2. UV-vis Analysis

**Figure 5:**
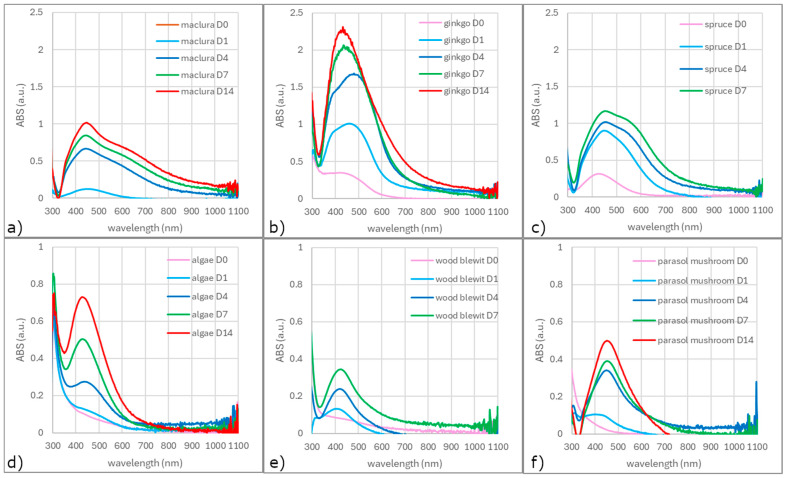
UV-vis spectra of colloidal silver solutions prepared by different extracts: maclura (**a**), ginkgo (**b**), spruce needles (**c**), *Ch. kessleri* (**d**), *Collybia nuda* (wood blewit) (**e**), and *Macrolepiota procera* (parasol mushroom) (**f**).

Not all extracts reduce silver nanoparticles immediately after being added to the AgNO_3_ solution (Figure 5). Just the ginkgo and spruce needles showed (on D0) small peaks at 420 and 426 nm, respectively, which is typical for silver spherical nanoparticles [22,23]. The AgNPs prepared by the maclura and fungi extracts showed small peaks at D1, and those for the alga peaked up to D4. This indicates that some extracts contain more effective reducing agents than others, which causes differences in the synthesis rate and its efficiency. Differences in the shape of the spectra are also evident.

Generally, a symmetrical and narrow spectrum with an ABS_max_ close to the wavelength of 430 nm indicates the presence of spherical nanoparticles. Such a spectrum shows a colloid prepared using an alga extract. The broadening of the absorption band indicates an increase in the interval of the size distribution and the presence of more quasi-spherical nanoparticles (extracts of both mushrooms).

Naghmachi et al. successfully synthesized AgNPs (spherical, with an average diameter of 32 nm) by extracting *Pistacia terebinthus* [24]. The received ABS_max_ was near 400 nm and, over time (24 h), moved to 430 nm and significantly increased. The authors associated the increase in ABS_max_ with the synthesis process rate (The concentration of AgNPs gradually increased, and after 24 h, the spectrum became stable.) and the red shift of ABS_max_ with an increase in the nanoparticle size. Such behavior of UV-vis spectra was also observed by Sharifi-Rad et al. [25]. Their findings and conclusions agree with our results.

A second peak or just a shoulder can indicate the presence of shapes other than spheres. The presence of the second peak can also indicate the presence of spherical nanoparticles of different sizes. But in our case, we suppose that the presence of differently shaped nanoparticles is because there is no distinct peak and just a shoulder. The shape of the spectra of the colloids prepared using extracts from maclura and spruce indicate the presence of spherical and non-spherical or differently shaped nanoparticles (because the second peak—or rather a shoulder—at approximately 550–600 nm is presented). The shape of the spectrum of the colloid prepared using ginkgo extract changed its shape over time, indicating a possible dynamic change in the shape of the nanoparticles.

**Figure 6:**
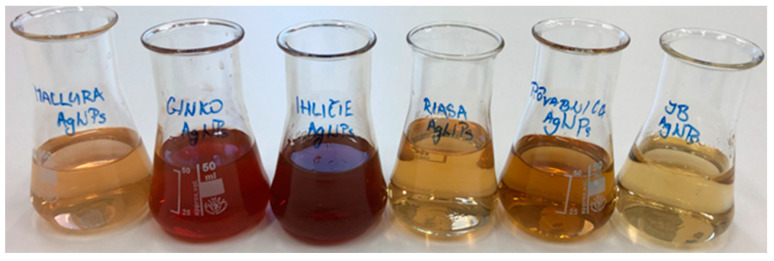
AgNP colloids prepared by the biological method on the seventh day (D7): maclura, ginkgo, spruce needles, *Ch. kessleri*, *Collybia nuda*, and *Macrolepiota procera* (from left to right).

The ABS_max_ of all spectra increased over time, and the colors of all solutions darkened (Figure 6). Based on the change in color and the increase in ABS_max_, it is possible to assume an increase in the volume of nanoparticles in the colloids. The relationship between the ABS_max_ value and the concentration of nanoparticles in the solution follows from the Beer—Lambert law:

(1) $$I=I_{0}\cdot 10^{\varepsilon_{\lambda }\cdot c\cdot l}$$

where *I*_0_ is the intensity of light traveling into an absorbing body (W); *I* is the intensity of light traveling out of an absorbing body (W); *c* is the concentration of the solution (in g·l^−1^); *ε_λ_* is the molar absorption coefficient at a wavelength λ (in dm^3^.g^−1^·cm^−1^); *l* is the length of the absorbing material (in cm); and *A* is the absorbance.

This law can be converted in terms of absorbance:

*A* = *ε_λ_·c·l* (2)

Based on this, we can conclude that the increase in ABS_max_ is proportional to the increase in the concentration of nanoparticles in the solution.

The differences in synthesis rate and yield are related to the composition of the extract and probably the pH of the solution as well. The alga and both mushrooms contained, according to the UV-vis spectra, strong stabilizing agents that stabilized the synthe sized nanoparticles, and their shape and size did not change over time. On the other hand, maclura, ginkgo, and spruce reduced the silver almost immediately, but spherical nanoparticles and nanoparticles of other shapes were expected.

### 3.3. TEM Analysis

**Figure 7:**
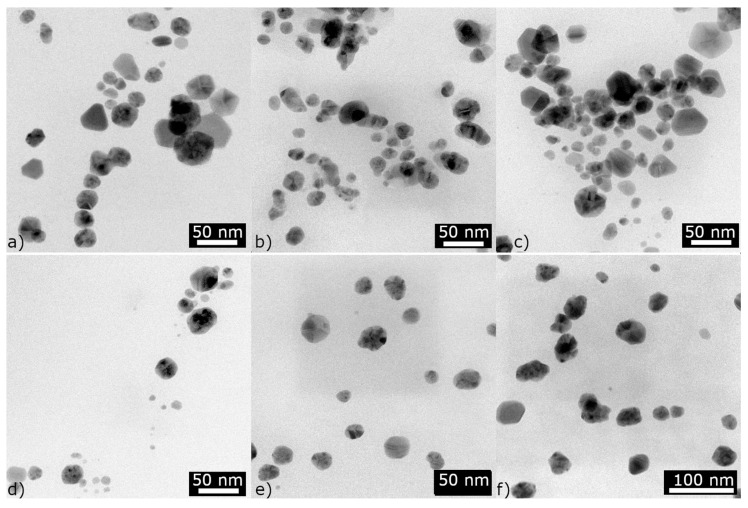
TEM images of AgNPs synthesized (D4) by maclura (**a**), ginkgo (**b**), spruce (**c**), *Ch. kessleri* (**d**), *Collybia nuda* (**e**), and *Macrolepiota procera* (**f**).

**Figure 8:**
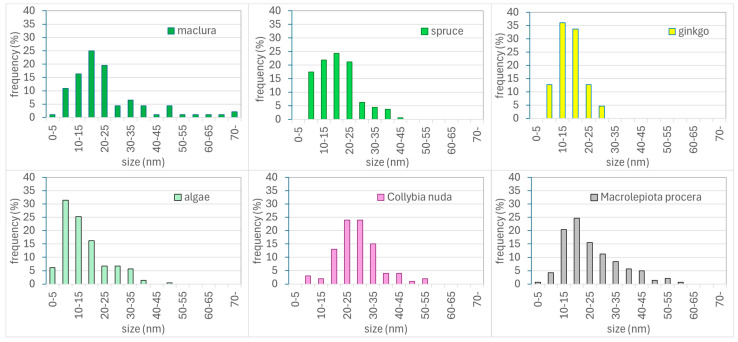
Histograms of the size distribution of AgNPs synthesized by maclura, spruce, ginkgo, algae, *Collybia nuda*, and *Macrolepiota procera*.

The conclusions from the UV-vis spectrophotometry were confirmed by TEM images (Figure 7). The average sizes of the spherical nanoparticles were 21 nm and 16 nm for maclura and spruce, respectively (Figure 7a,c). The size distribution histograms for the AgNPs synthesized by the maclura and spruce extracts are in Figure 8, where maclura shows a wider size distribution interval and nanoparticles with sizes up to 70 nm can be observed. The spruce extract synthesized nanoparticles in the interval from 5 to 45 nm, and just 18% of the AgNPs were larger than 25 nm.

**Figure 9:**
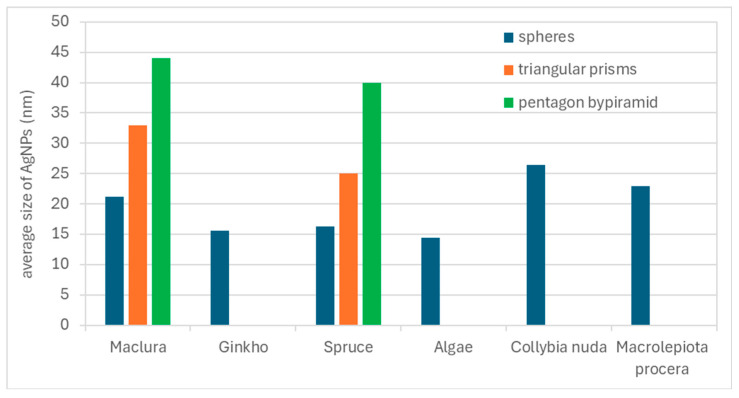
The average size of the AgNPs (D4).

The nanoparticles prepared with the extracts of maclura (Figure 7a) and spruce (Figure 7c, aside from spherical nanoparticles, contained polyhedrons (hexagonal and pentagon bi-pyramids) with sizes of 44 nm and 40 nm and prismatic triangular nanoparticles with sizes of around 33 nm and 25 nm for the macula and spruce needles, respectively (Figure 9). The size of the triangular nanoparticles was determined as an average of three triangular heights. The pentagons were measured in two perpendicular directions, and the average size was calculated.

The presence of nanoparticles with different sizes and shapes in colloids prepared by the biological method was also confirmed by Handayani et al.. The authors observed the effect of pH on the change in the shape and size of AgNPs and found that at pH levels of 5 and 11, it is possible to obtain not only spherical but also triangular and pentagonal nanoparticles [17]. They attributed this to the fact that synthesizing spherical nanoparticles with small dimensions requires faster reactions, which can be ensured by low pH values (i.e., below five). However, based on our results, not only is the pH level a determining factor but also the type and composition of the extract.

Only spherical nanoparticles (stable, with no change over time) were confirmed in the colloids prepared by algae and fungi extracts. The average size of these nanoparticles was 14 nm, 26 nm, and 23 nm for the algae, *Collybia nuda*, and *Macrolepiota procera*, respectively (Figure 7d–f), and the size distribution histograms are in Figure 8. The leaves of ginkgo synthesized spherical nanoparticles with an average size of 16 nm and with a narrower size distribution interval (5–30 nm) (Figure 8).

**Figure 10:**
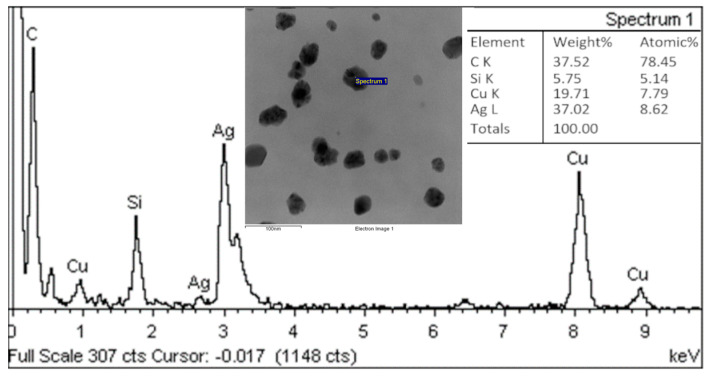
EDS analysis of AgNPs prepared by extract of *Collybia nuda.*

EDS analysis of the nanoparticles was carried out on all samples, and silver was confirmed in all samples. As an example, we present the results of the analysis of nanoparticles prepared using an extract from *Collybia nuda* (Figure 10). The presence of silver but also other elements such as Cu and Si is obvious, the Cu signal was caused by the copper grid, which was used in sample preparation, and Si is an impurity from the carbon film. Table S1 proves the presence of silver in the nanoparticles and shows the chemical compositions of the measured samples.

### 3.4. FTIR Analysis

As the procedure of AgNP synthesis was the same for all extracts, we conclude that differences in the compositions of the extracts caused the differences in the shapes of the prepared nanoparticles and synthesis efficiency. All extracts and silver nanoparticle colloids were analyzed by FTIR spectroscopy. The infrared radiation impacted the atomic vibrations of a molecule in the sample, resulting in the specific absorption or transmission of energy. FTIR is used to determine organic compounds, including chemical bonding, as well as organic content [26,27,28,29,30,31,32,33,34,35,36,37,38,39].

Figure 10 compares the FTIR analyses of the extracts and colloids after nanoparticle synthesis. Post-synthesis FTIR analysis was performed to identify possible interactions between silver and bioactive molecules that may be responsible for the synthesis and stabilization of silver nanoparticles.

#### 3.4.1. *Maclura pomifera* Fruit

**Figure 11:**
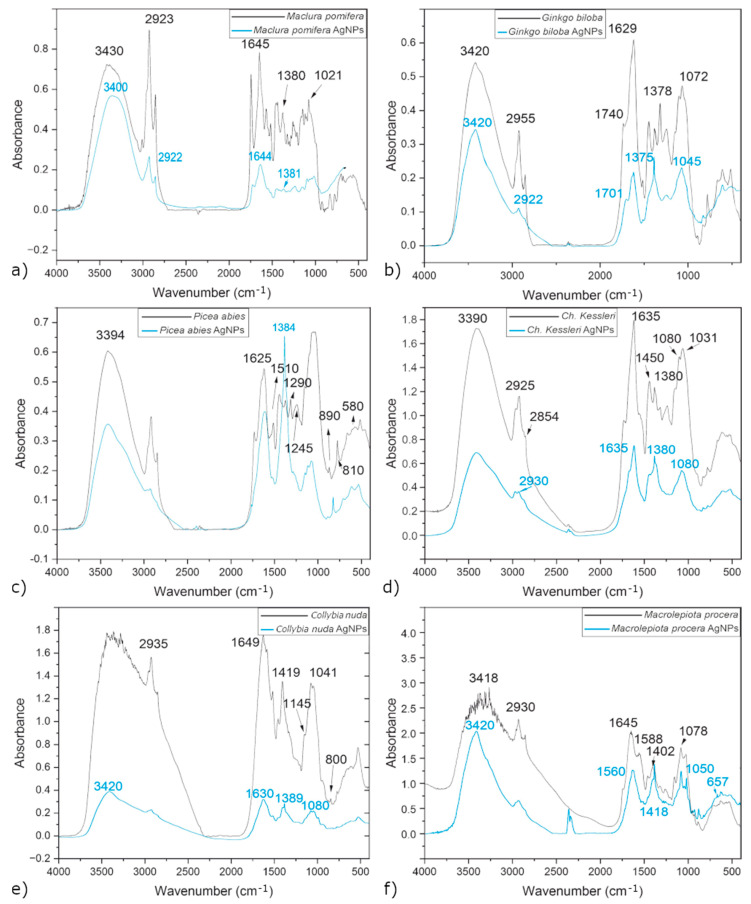
FTIR spectra of extracts and colloids after AgNP synthesis: maclura (**a**), ginkgo (**b**), spruce needle (**c**), *Ch. kessleri* (**d**), *Collybia nuda* (**e**), and *Macrolepiota procera* (**f**).

The FTIR spectra of the maclura extract and the colloids after synthesis are in Figure 11a. The broad and strong absorption band at 3429–3434 cm^−1^ is assigned to hydroxyl (-OH) or amine (-NH) groups [26] and corresponds to the presence of phenolics or amino acids. The bands at 2923 and 2922 cm^−1^ can be attributed to the -CH group, including the aliphatic -CH_3_ and -CH_2_ stretching vibration [27]. The bands observed at 1645 and 1644 cm^−1^ are assigned to the stretching vibration of the carbonyl group (C=O), corresponding to the amides (NH_2_) [28]. The absorbance at around 1380 and 1381 cm^−1^ can be assigned to the primary amine group (-NH_2_) or the bending vibrations of the -CH group. The absorbance band observed at 1021 cm^−1^ can be designated as the C-O stretching of polysaccharides [29].

Changes in absorbance between the maclura extract and the sample after AgNP synthesis show that mainly the carbonyl (C=O), hydroxyl (-OH), and amine (-NH) groups of the extract are involved in AgNPs synthesis. Their change presents the reduction and stabilization of silver nanoparticles, caused by the interaction between the hydroxyl group of phenols (probably pomiferin) and silver ions or by coordination between the nitrogen of the amide group and silver ions.

Pomiferin is one of the predominant isoflavones in maclura fruit. This isoflavone is also a powerful antioxidant similar to other important flavonoid compounds such as catechin, rutin, and quercetin. According to Azizian-Shermeh [30], we can assume that pomiferin, as a strong antioxidant, could reduce Ag+ silver ions and transform them into silver nanoparticles. FTIR analysis confirmed only the presence of bands corresponding to the functional groups present in flavonoids.

It was also demonstrated that proteins can bind to nanoparticle surfaces, which shows that proteins can form a layer covering metal nanoparticles that stabilize AgNPs and thus prevent their agglomeration [30].

#### 3.4.2. *Ginkgo biloba* Leaves

Bioactive compounds such as flavonoids, terpenoids, phenols, glycosides, and ginkgolides are typical for ginkgo [10,11]. In the ginkgo spectrum (Figure 11b), the presence of -CH valence vibrations of the functional groups -CH_3_ or -CH_2_ at 2955 cm^−1^, -OH valence vibrations at 3420 cm^−1^, C-O vibrations of the carboxyl group C=O at 1378 cm^−1^ and 1740 cm^−1^, and alkylphenols at 1072 cm^−1^ were confirmed. The bands at the wavelength of 1629 cm^−1^ are for the -OH, C-O, and C=O groups. These functional groups are associated with bioactive molecules such as phenols, flavonoids, terpenoids, organic acids, and ginkgolides.

Most of the bands after AgNP synthesis were absent or shifted due to the bioreduction process compared with the spectrum of the extract. The most significant shift (to lower wavelengths) after the reduction of silver nanoparticles was observed at the bands of 1619, 1701, 1045, and 2922 cm^−1^. Such a change was also observed by other authors, such as Ezzat et al. and Beek et al. [10,11]. These authors suggested that bioactive molecules such as phenols, flavonoids, terpenoids, organic acids, and ginkgolides, which contain the mentioned functional groups and which participated in the reduction, are responsible for the reduction of Ag^+^ ions to Ag^0^.

#### 3.4.3. Spruce Needles

The FTIR spectra of the spruce needle extract and the AgNP colloids are in Figure 11c. Strong bands at 3394 cm^−1^ and 1605 cm^−1^ were assigned to -OH bonds from phenolic compounds and to the carbonyl group. The infrared absorption spectra of the spruce needles also show absorption bands such as alkyl nitrites (1640–1610 cm^−1^), secondary alkylamines (1510 cm^−1^), esters of unsaturated aromatic acids (1290 cm^−1^), secondary amides (1245 cm^−1^), benzene derivatives (890 cm^−1^) and sulfonic acids (820 and 580 cm^−1^), which were also confirmed in other works [31,32].

In the spectrum of the sample after AgNP synthesis, an intense band appeared at a wavelength of 1384 cm^−1^, which corresponds to the asymmetric valence vibration of the carboxylate ion (COO-), a newly formed group after the reduction of silver.

#### 3.4.4. Green Alga *Ch. kessleri*

The FTIR analysis of the extract prepared from *Ch. kessleri* is shown in Figure 11d. The absorption band near 3390 cm^−1^, denoted as -OH, confirms the presence of a strong alcohol group. The absorption bands between 2854 and 3010 cm^−1^ correspond to the -CH valence vibration in the CH_3_ and CH_2_ groups. The absorption bands of the proteins in *Ch. kessleri* were characterized by strong vibrations at 1635 cm^−1^ (amide I). This band was mainly due to the C=O valence vibrations and the combination of -NH and -CH valence vibrations in the amide complexes. The lipids and carbohydrates were characterized by strong C-H vibrations at a wavelength of 2925 cm^−1^ and the C-O-C of polysaccharides at 1080 cm^−1^ and 1031 cm^−1^, respectively [33,34]. A significant absorption band at 1450 cm^−1^ confirms the vibrations of the methyl groups of the protein [35]. The band at about 1380 cm^−1^ is related to the C=O stretching from carboxylate [36].

After the synthesis of AgNPs, a significant decrease in the bands around 1635 cm^−1^ can be observed. These bands (amide I and proteins of the methyl groups) were clearly identified in the extract but less significant in the post-synthesis sample. We can assume that the decrease in the intensity of the bands occurred due to the synthesis of AgNPs. The absorption band at about 1380 cm^−1^, which appeared after AgNP synthesis, was significantly sharper. This peak is a significant increase in intensity compared with the band in the extract. The most probable reason for this is that after adding the extracts to the AgNO_3_ solution, the aldehyde group present in the saccharide was oxidized to carboxylic acid by Ag^+^, and therefore the band at 1380 cm^−1^ increased.

#### 3.4.5. *Collybia nuda*

Strong and broad bands at 3371 cm^−1^ correspond to the valence vibration of the hydroxyl groups (Figure 11e). The valence vibration of glycosylalkyl (-CH) reached a band at 2935 cm^−1^. The C=O valence vibration created an absorption band of 1649 cm^−1^. The absorption band at 1419 cm^−1^ corresponds to the C-O valence vibration of the carboxyl group. The absorption band of the S=O bond and the sulfate symmetric group was at 1145 cm^−1^. The band at the wavelength of 1079 cm^−1^ was due to the vibrations of the C-O-C and C-O-H groups. We can assign the absorption band at 1041 cm^−1^ to the vibration of the pyranose ring, and the absorption band at 800 cm^−1^ corresponds to the vibration of the C-H group of the α-pyran ring [37,38]. Shu et al. also confirmed the presence of carboxyl and sulfate-containing polysaccharides and, in addition, acid polysaccharides of the furanose and pyranose types [38]. The rather complex composition of the fruiting body of *Collybia nuda* confirms Pinto et al., who demonstrated the presence of vitamin B1, triterpenoids (-OH), sterols (-OH), proteins (-NH), carbohydrates (-CH), fatty acids (stearic, oleic, and linoleic), and organohalogens [39]. Regarding the spectrum of the sample after AgNP synthesis, there was a decrease in all band intensities.

#### 3.4.6. *Macrolepiota procera*

In the FTIR spectrum of *Macrolepiota procera* (Figure 11f), the (-OH), (-CH), (-NH), and (C-C) bands presented at 3418 cm^−1^, 2930 cm^−1^, 1588 cm^−1^, and 1402 cm^−1^, were assigned to alcohol, amine, aromatic, and alkyne, respectively. The band at 1645 cm^−1^ corresponds to the primary amines, and the band at 1078 cm^−1^ was assigned to the aliphatic amines. This indicates that the amine group (-NH) acts as a capping agent for the synthesis of silver nanoparticles.

In the FTIR spectrum of the sample after AgNP synthesis, the bands at 3420, 1560, 1418, 1050, and 657 cm^−1^ correspond to alcohol (-OH), amine (-NH), aromatic (C-C), and alkyne (-CH), respectively. They were all slightly shifted compared with the extract, and their intensities also decreased.

The nanoparticle synthesis process involves several steps (phases) and follows the classic LaMeer model. The initial activation phase involves the reduction of silver ions by plant biomolecules and nucleation of the reduced metal atoms. During the second step, growth of the nucleus occurs as a spontaneous aggregation of adjacent nanoparticles to form larger-sized particles. In the final step, capping of the aggregated particles takes place, giving the final shape to the nanoparticles. In plant-mediated synthesis, phytochemicals and biomolecules play a key role in Ag^+^ ion reduction. The primary phytochemicals involved are terpenoids, polyphenols, carbohydrates, alkaloids, phenolic acids, and proteins [40]. Commonly, these reducing agents and other metabolites present in the plant extract also work as capping and stabilizing agents [41]. This gives an additional advantage to plant-mediated synthesis, as no additional stabilizing agents are required.

From the above analysis, we can see that some bands and structures of all samples were similar, and it is evident that after synthesis, some bands decreased, shifted, or even disappeared. Such changes indicate biochemicals that are responsible for the reduction and stabilization of AgNPs.

For example, the -OH group can indicate the presence of water, alcohol, and phenols, but it is also present in amino acids. The exact biochemical substance can be determined by the presence of other specific bands. On the other hand, some extracts differ in some bands. For example, the functional groups typical for flavonoids, terpenes, or phenols are present in maclura and spruce needles, but polyphenols, proteins, and carbohydrates are more typical for ginkgo, algae, *Collybia nuda,* and *Macrolepiota procera*. Our results show that plants that contain essential oils or resins (spruce resin) can synthesize non-spherical nanoparticles (triangles and polygons) in addition to spherical one (Figure 7a,c). Some authors mentioned that proteins [9,30] and carbohydrates [42] act as effective stabilizers. In such a case, it is possible to expect effective stabilization of nanoparticles (algae, *Collybia nuda,* and *Macrolepiota procera*), and presumably, just spherical nanoparticles will form (Figure 7d–f).

The exact determination of the phytochemicals that are responsible for reduction and stabilization is quite difficult because often, the same phytochemical contributes to both reduction and stabilization. But, from the experiments it is clear that it is also possible to prepare them through biological synthesis. Using a suitable plant, even nanoparticles of a shape other than a spherical one can be formed. However, it is necessary to choose not only a suitable plant (or part of it) but also to separate the substances that affect the shape. We assume that the substances that influence the shape of nanoparticles include mainly resins, the main compounds of which are terpenes and phenols. It is possible to adjust the composition of the spruce needle extract, for example, by fractional distillation, by preparing the extract at a different temperature than the ambient temperature, or by multi-stage extraction.

## 4. Conclusions

We successfully prepared silver nanoparticles through a biological method. The silver nanoparticles were synthesized by extracts which were extracted from various parts of plants. Fruit of the maclura tree, spruce and ginkgo needles, green algae, and mushrooms, namely *Collybia nuda* and *Macrolepiota procera*, were used. The compositions of the extracts and colloids of AgNPs were analyzed using FTIR analysis. FTIR is used to determine organic compounds, including chemical bonding as well as the organic content. It was proven that the prepared extracts contained reducing and stabilizing substances. The prepared nanoparticles were mainly of spherical and quasi-spherical shapes (size range: 10–25 nm), but it was found that some plants were also able to synthesize nanoparticles of other shapes: triangular prisms or even polygons. Until now, it was generally known that it is possible to prepare only spherical and quasi-spherical nanoparticles through green synthesis, but our experiment proved that it is possible to prepare shapes other than spherical ones. FTIR analysis confirmed vibrational bands that could refer to flavonoids, terpenes, and phenols, the main compounds of resins, which are probably mainly responsible for forming differently shaped nanoparticles.

Such shapes were not dominant, but by adjusting the compositions of the extracts, it would probably be possible to prepare the desired shape in a greater amount. These findings need further confirmation. Therefore, this will be the subject of further research.

## Figures and Tables

**Table 1:** The amount of biomass and deionized water used for extract preparation and the process temperature.

Plant	Part	Weight (g)	H_2_O (mL)	Temperature (°C)
*Maclura pomifera*	fruit	0.88	25	80
*Ginkgo biloba*	needle	2.50	25	80
*Picea abies*	needle	0.75	25	80
*Ch. kessleri*	alga	1	25	80
*Collybia nuda*	mushroom	0.8	25	80
*Macrolepiota procera*	mushroom	0.62	25	80

## Data Availability

Data are contained within the article and Supplementary Materials.

## Supplementary Materials

The following supporting information can be downloaded at https://www.mdpi.com/article/10.3390/ma17102252/s1. Table S1: The chemical composition of measured nanoparticles prepared by different extracts.
