# Supplementary material for: Impact of Plant Extract Phytochemicals on the Synthesis of Silver Nanoparticles
Source: Materials (Basel). 2024 May 10;17(10):2252. doi: 10.3390/ma17102252 (PMC11123381; doi:10.3390/ma17102252)
Supplement: Supplementary file 1 [file materials-17-02252-s001.zip › materials-2992385-supplementary.pdf]

Table 1. The chemical composition of measured nanoparticles prepared by different extracts.

wt.%	maclura	ginkgo	spruce needles	*Ch. kessleri*	*Collybia nuda*	*Macrolepiota procera*
C	35.01	42.93	38.4	47.16	37.52	28.7
Ag	48.94	35.93	38.7	30.6	37.02	52.7
Si	5.81	3.75	8.6	10.94	5.75	11.8
Cu	10.24	17.39	14.3	11.3	19.71	6.8
